# Combined linkage and association mapping reveals candidates for *Scmv1*, a major locus involved in resistance to sugarcane mosaic virus (SCMV) in maize

**DOI:** 10.1186/1471-2229-13-162

**Published:** 2013-10-18

**Authors:** Yongfu Tao, Lu Jiang, Qingqing Liu, Yan Zhang, Rui Zhang, Christina Roenn Ingvardsen, Ursula Karoline Frei, Baobao Wang, Jinsheng Lai, Thomas Lübberstedt, Mingliang Xu

**Affiliations:** 1 National Maize Improvement Center, China Agricultural University, 2 West Yuanmingyuan Road, Beijing 100193, People’s Republic of China; 2 Department of Agronomy, Iowa State University, 1204 Agronomy Hall, Ames, Iowa 50011, USA; 3 Research Center Flakkebjerg, University of Aarhus, 4200, Slagelse, Denmark

**Keywords:** Maize, SCMV, QTL, Fine-mapping, Association mapping

## Abstract

**Background:**

Sugarcane mosaic virus (SCMV) disease causes substantial losses of grain yield and forage biomass in susceptible maize cultivars. Maize resistance to SCMV is associated with two dominant genes, *Scmv1* and *Scmv2*, which are located on the short arm of chromosome 6 and near the centromere region of chromosome 3, respectively. We combined both linkage and association mapping to identify positional candidate genes for *Scmv1*.

**Results:**

*Scmv1* was fine-mapped in a segregating population derived from near-isogenic lines and further validated and fine-mapped using two recombinant inbred line populations. The combined results assigned the *Scmv1* locus to a 59.21-kb interval, and candidate genes within this region were predicted based on the publicly available B73 sequence. None of three predicted genes that are possibly involved in the disease resistance response are similar to receptor-like resistance genes. Candidate gene–based association mapping was conducted using a panel of 94 inbred lines with variable resistance to SCMV. A presence/absence variation (PAV) in the *Scmv1* region and two polymorphic sites around the *Zmtrx-h* gene were significantly associated with SCMV resistance.

**Conclusion:**

Combined linkage and association mapping pinpoints *Zmtrx-h* as the most likely positional candidate gene for *Scmv1*. These results pave the way towards cloning of *Scmv1* and facilitate marker-assisted selection for potyvirus resistance in maize.

## Background

Sugarcane mosaic virus is one of the most prevalent viral pathogens, causing significant global losses in maize grain and forage yields [1], especially in China and Germany [1,2]. A recent report of SCMV in Poland and Central France revealed that the incidence of SCMV continues to increase [3,4]. The diagnostic symptoms of infection with SCMV include stunting, chlorosis, and reduction in plant weight and grain yield [1,5]. SCMV, formerly denoted as maize dwarf mosaic virus (MDMV) strain B, and belongs to the family *Potyviridae*[5]. Other members of the family *Potyviridae* that also infect maize include MDMV, wheat streak mosaic virus (WSMV), Johnson grass mosaic virus (JGMV), sorghum mosaic virus (SrMV), and Zea mosaic virus (ZeMV) [6,7]. Control of SCMV by agronomic and chemical means is ineffective owing to the non-persistent transmission mode of the virus by aphid vectors. Thus, deployment of resistant varieties is the most important way to prevent yield losses, which is based on screening and identification of resistant germplasm [8].

U.S. inbred line Pa405 shows complete resistance to MDMV and SCMV under both field and greenhouse conditions after artificial inoculation [9]. In Europe, a collection of 122 early maturing maize inbred lines was screened for resistance against SCMV, resulting in the identification of three lines, D21, D32, and FAP1360A, that displayed complete resistance to SCMV [6]. In China, many elite inbred lines display complete resistance when challenged with SCMV inoculum [2,10]. Analysis of quantitative trait loci (QTL) in a cross between the resistant line D32 and susceptible line D145 revealed two major dominant genes on chromosomes 3 and 6, and three minor QTL on chromosomes 1, 5, and 10 [11,12]. High-resolution mapping using progeny from the cross between FAP1360A (completely resistant to SCMV) and F7 (highly susceptible to SCMV) confirmed that *Scmv1* (on the short arm of chromosome 6) and *Scmv2* (near the centromere of chromosome 3) are crucial for maize resistance to SCMV [13]. Similar mapping results have been reported in several independent experiments [8,14,15]. The *Scmv1* locus has also been detected in a resistant Chinese line, Huangzao4, together with additional QTL on chromosomes 1, 3, 5, and 10 [14]. In the progeny of Siyi×Mo17, two major genes were mapped on chromosome 3 (bin 3.04/05) and chromosome 6 (bin 6.00/01), respectively [8]. By using an F_9_-derived immortalized RIL population obtained from the cross of Huangzao4×Mo17, a major QTL was detected between chromosome bins 6.00 and 6.01, accounting for 50.0% of the total phenotypic variation and decreasing the disease index by 27.9% [16]. The loci on chromosomes 3 and 6 have also been confirmed in tropical maize germplasm from Brazil [15]. With the aid of the whole-genome B73 sequence, the *Scmv2* region was fine-mapped to a region of 0.28 cM, covering a physical distance of 1,342.6 kb. Four predicted genes possibly involved in virus movement are likely candidates for *Scmv2*[17]. Existence of two closely linked resistance genes within the *Scmv1* region has been postulated [18,19]. Interestingly, several QTL that confer resistance to various viruses all map to the same region on chromosome 6 [20]. It is currently uncertain, whether a single pleitropic *Scmv1* gene confers resistance to different viruses, or distinct resistance genes are clustered in this region.

Even though diverse mapping populations have been used, all studies consistently identified the *Scmv1* and *Scmv2* genomic regions as being critical to confer resistance to SCMV. In this study, we first conducted the fine-mapping of *Scmv1* using a large isogenic mapping population previously shown to segregate solely for *Scmv1*[13,18,19,21,22]. Second, we employed two RIL populations from elite parental lines with contrasting resistance to SCMV to confirm and further fine map the *Scmv1* locus. Third, we identified a limited number of positional *Scmv1* candidate genes based on the combined fine-mapping results. Finally, we conducted candidate gene-based association mapping to characterize the QTL region and candidate genes using a panel of 94 maize inbred lines with variable resistance to SCMV.

## Results

### Fine-mapping of *Scmv1* in a near-isogenic F_2_ population

**Figure 1:**
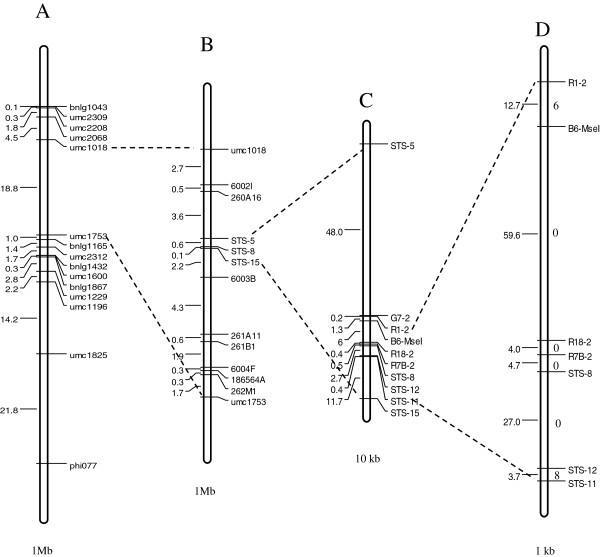
**Diagram of the sequential fine-mapping process. (A)** The physical map of 17 polymorphic SSR markers available from MaizeGDB (http://www.maizegdb.org/) in the *Scmv1* genome region, 2 of the 17 markers with ambiguous location were deleted. By assaying 177 unselected F_2_ plants, *Scmv1* was narrowed to an 18.8 Mb interval flanked by SSRs umc1018 and umc1573. **(B)** The physical map of the *Scmv1* region after fine-mapping with 28 recombinants and 13 new developed markers. **(C)**, **(D)** The final physical maps of *Scmv1*, estimated by genotyping 15 recombinants between STS-15 and STS-5 using eight newly developed markers. The number on the left side of the chromosome segments correspond to the physical distance of AGPv2, the units of A, B, C and D were 1 Mb, 1 Mb, 10 kb, 1 kb, respectively. The numerals on the right side of the chromosome segments represent the number of recombinants between two flanking markers.

Of 177 unselected F_2_ plants, 50 exhibited typical symptoms of SCMV infection within 2–3 weeks after the first inoculation. The numbers of resistant and susceptible plants segregated in a 3:1 ratio (χ^2^ = 0.83 < χ^2^_0.01, 1_), in agreement with inheritance of a single dominant gene. Using the 17 polymorphic simple sequence repeat (SSR) markers available from the Maize Genetics and Genomics Database (http://www.maizegdb.org/) (Additional file 1), the *Scmv1* locus was mapped between umc1018 and umc1753, with a physical distance of 18.80 Mb according to the B73 physical map (Figure 1A). Owing to the lack of high-density markers, 28 recombinants between these two markers could not be resolved. Accordingly, we developed 11 new markers (Table 1) based on the public maize Bacterial Artificial Chromosome (BAC) sequences accessible in GenBank at the National Center for Biotechnology Information (http://www.ncbi.nlm.nih.gov). We used these 11 new markers to resolve the 28 recombinants. Subsequent marker-phenotype analysis enabled us to progressively refine the *Scmv1* region into a 710.97-kb interval between STS-5 and STS-15 (Figure 1B), with still two recombinants in between those two markers. Thereafter, the markers STS-5 and STS-15 were used to genotype 510 F_2_ susceptible plants, resulting in 13 new recombinants. Within the STS-5/STS-15 interval, single/low copy sequences were exploited to develop seven additional markers (Table 1). These markers were used to genotype 15 recombinants, which allowed us to delimit the *Scmv1* locus into a 112.39-kb interval flanked by the two closest markers, R1-2 and STS-11 (Figures 1C and 1D). There were still six recombinants to the left and eight recombinants to the right side of the *Scmv1* locus (Figure 1D). Comparison with the B73 physical map indicated that the two closest markers, R1-2 and the STS-11, reside on the adjacent BACs in GenBank: b0129G15 and b0598N23, respectively.

**Table 1:** **New markers developed for fine mapping of the *Scmv1 *** **locus**

**Name**	**BAC**	**Forward primer**	**Reverse primer**	**Type**	**Restriction enzymes**
6002I	c0288C01	GTTAGAACGTCTGCGCCTGT	TCCGTGCTGCGTAGTTACCT	STS	
STS-5	c0149g11	GCAGGAGAAGAATCGGAGG	ACAAGAACAAGACAGCAGCG	STS	
G7-2	b0129G15	AGTGTTCGTACCATGAGGTC	AATCCAGCCTCAGTAGAGTC	STS	
R1-2	b0129G15	GCGTAGCAATATCCGTGTCA	TTCTGGTGTGAGCTTGAGCC	STS	
R18-2	b0598N23	GATCTCGCAATCGTTGTAGC	CTCTCAATGCGGTAACCATC	STS	
STS-8	b0129G15	ACCGATTATGTTCCATTGGC	GGTCCGACGCTCACTTCC	STS	
STS-11	b0598N23	CAATAGACGTCGGACGAATG	ATGACATGCAACTACAGGCG	STS	
STS-12	b0598N23	CTAACAAGCATGACGATCCG	AATTGCATCGATAAGCCACC	STS	
STS-15	b0606n15	CTATTGGGCTTCCTTGTTCG	TATTCCTGTAGGCGACCTGG	STS	
261B1	c0042B13	AGCTTCCAAGTGTCCTTAGC	GCATCCTGGCTCAGCAATAC	STS	
6003B	b0191N03	CTGATGACTGCCACCATAGC	AACACGCTCGTGAGCAATAC	STS	
186564A	b009O12	GTTCACGTTTCCCATGCTG	GCCACGTCGAACAACCTTAT	SSR	
261A11	c0361N19	GAGAAGGCGAGAACGCATTG	AAGAGGCTACCGTAGGCGTC	SSR	
6004F	b0390N08	CGAGCTACTCAGCGTTGTCC	AAGAGGCTACCGTAGGCGTC	SSR	
R7B-2	b0598N23	CACCGGAATAGTAGACGCAC	AGCTCTACTCCACCGGAACA	SSR	
260A16	c0115G19	ATGTCTGCTGCCGTGAGTTC	CCTCCTGTCTGGTTGTCGTC	CAPS	*HaeIII*
B6-*Mse*I	b0129G15	GGATGAGAGGACTCTTGCAC	CTGCAAGCATGTCACAACA	CAPS	*MseI*
262M1	c0043A01	CAACTTCCATGACAGTGTCC	GTGTGATTCGGTGGCATAAC	CAPS	*MfeI*
RL3*	207B1-77	GGATACAGCACCAAGGTTGA	AAGAGCTGTACACAGACGCC	SNP	
SNP1*	207B1-77	GTAAGTGATAGGCGGAGTGG	AGGCTATCGTCGTGGATTGA	SNP	
SNP2*	207B1-77	CCACTCTTGCTTCATCCTCA	AGTGTGTACGTGACCTTGATCT	SNP	
SNP3*	207B1-77	AGATGGTGGTGAAGTGAAGG	CCTAGCAGGCTAGCACTGAT	SNP	
SNP4*	207B1-77	GATGGAGTGCCGATTGCTAG	GGTCAACGAAGCCGATATTG	SNP	
R6*	207B1-77	CACACACCACTTGCGATGTT	TCATGCGATGCCAGTGATAG	SNP	
2562F*	207B1-77	GCCGTATACAGGTCGAGCTT	GGAAGGAAGCCTAACTCGGA	SNP	
Trx-1*	207B1-77	CGATGCCGCCTAATATTCTC	GTAGCGGATCACGGATAACG	STS	
579P4*	207B1-77	AACGCGAGTGCAGTAAGTCT	TGTTCCACAATGCTTTATCC	CAPS	*AccII*
TC-4*	579B1-75	CAGAGGAACAACAACCACCA	GCACATTACGGTTGAGTTGG	SNP	
B-4*	579B1-75	TACAAGTCAGGAGGTCCGGT	CTGGATGGTGTTGTGTCGAG	SSR	

*: Markers used for validation and further mapping in RILs, and these markers were developed based on the BAC sequences from 1145 inbred line.

### Validation of *Scmv1* in RILs

Given that the *Scmv1* region could not be narrowed further using the near-isogenic F_2_ population, we used two RIL populations with parental lines contrasting in resistance to SCMV to confirm and fine-map the *Scmv1* region. As both of the *Scmv1* and *Scmv2* loci are involved in maize resistance to SCMV, each RIL was investigated for its genotype at the *Scmv2* locus using the co-segregating marker DJF004 developed by Ingvardsen et al. [17]. Given that one of the *Scmv1* flanking markers, STS-11, failed to reveal any polymorphism between parents of each of the two RIL populations, we searched for an alternative marker around STS-11 and finally developed a new marker, B4, which is located approximately 103.75-kb downstream of STS11. The B4 marker, together with the R1-2 marker, were then used to jointly genotype all RILs at the *Scmv1* locus. For each RIL population, four sets of 30 RILs, which differed at either the *Scmv1* or *Scmv2* or both loci, were separately selected for phenotypic evaluation.

In the RIL population of Zheng58×Chang7-2, 28 of 30 RILs that share the same haplotype as Chang7-2 at both *Scmv1* and *Scmv2* loci (RR/RR) were highly resistant to SCMV. Of 30 RILs with the Chang7-2 haplotype at *Scmv1*, but the Zheng58 haplotype at *Scmv2* (SS/RR), only one RIL was resistant to SCMV. Another two sets of 30 RILs sharing the same haplotype as Zheng58 at the *Scmv1* locus (SS/SS and RR/SS) were all susceptible to SCMV. A similar result was observed in the RIL population of X178×HuangC, all 30 RILs sharing the same haplotype as X178 at both *Scmv1* and *Scmv2* loci (RR/RR) displayed high resistance to SCMV. Of 30 RILs with the X178 haplotype at *Scmv1* and HuangC haplotype at *Scmv2* (SS/RR), only four RILs were resistant to SCMV. Another two sets of 30 RILs which share the same haplotype as HuangC at the *Scmv1* locus (SS/SS or RR/SS) were all susceptible to SCMV. These findings indicate that the presence of both *Scmv1* and *Scmv2* loci are prerequisite for SCMV resistance as described in previous reports [13]. The significant difference (*P* < 0.01) in resistance percentages between RILs differing in haplotypes at both *Scmv1* and *Scmv2* loci suggested the presence of the same resistance loci in both RIL populations (Table 2).

**Table 2:** **Confirmation of the *Scmv1 *** **and *Scmv2 *** **regions in two RIL populations**

		**Genotype**				***P*-value**
		**SS/SS**	**SS/RR**	**RR/SS**	**RR/RR**	
Zheng58×Chang7-2						
	Resistant RILs	0	1	0	28	<0.01
	Susceptible RILs	30	29	30	2	
HuangC×X178						
	Resistant RILs	0	4	0	30	<0.01
	Susceptible RILs	30	26	30	0	

The haplotype at the *Scmv1* locus was detected by its flanked SSR markers R1-2 and B4; while the haplotype at the *Scmv2* locus was identified by its co-segregating marker DJF004. The genotype SS/SS means both of the *Scmv1* and *Scmv2* loci are the same as Zheng58 or HuangC; RR/RR means both loci are the same as Chang7-2 or X178; RR/SS means *Scmv2* and *Scmv1*are the same as Chang7-2/X178 and Zheng58/HuangC, respectively; while SS/RR means *Scmv2* and *Scmv1* are the same as Zheng58/HuangC and Chang7-2/X178, respectively.

*P*-value and χ^2^ were calculated by SAS 9.1 using two-way ANOVA.

### Further fine-mapping of *Scmv1* in RILs

Given the validation result, the flanking markers B4 and R1-2 were used to screen for recombinants within the 215.47-kb *Scmv1* interval. Eight and nine recombinants were detected in the RIL populations Zheng58×Chang7-2 and X178×HuangC, respectively. For the *Scmv2* locus, a co-segregating marker DFJ004 was used to determine the genotype for each RIL. All recombinants were repeatedly investigated for their resistance to SCMV under both greenhouse and field conditions. On the other hand, through multiple comparisons of three resistant inbred lines, namely 1145, FAP1360A, and Huangzao4, and one susceptible B73 sequences within the B4/R1-2 interval, ten markers were developed to resolve the 17 recombinants.

**Figure 2:**
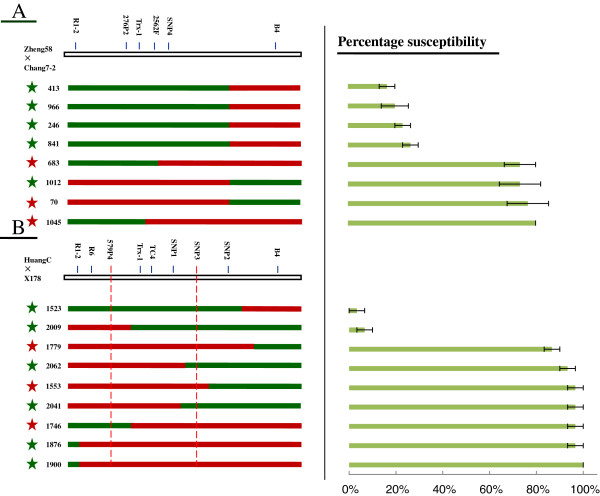
**Further fine-mapping of *Scmv1 *in two RIL populations.** The genetic structure for each recombinant type is depicted as green and red rectangles, (green symbolizes the fragment from resistant parents, chang7-2 or X178; red symbolizes the fragment from susceptible parents, Zheng58 or HuangC). The star represents allele at the *Scmv2* locus on chromosome 3 (green symbolizes resistance allele from Chang7-2 or X178; red symbolizes susceptibility allele from Zheng58 or HuangC). The light-green bars on the right represent the average percentage of plants with SCMV symptoms based on three independent experiments in greenhouse. **A)** The *Scmv1* locus can be delimited to a 215.47-kb region in RIL population Zheng58×Chang7-2. **B)** The *Scmv1* locus can be delimited to a 59.21-kb region flanked by markers 579P4 and SNP3 (in red dash-dot lines) in RIL population HuangC×X178.

Of the eight recombinants screened from the RIL population of Zheng58×Chang7-2, four RILs were as resistant to SCMV as the resistant parent Chang7-2, whereas another four RILs were as susceptible to SCMV as the susceptible parent Zheng58. The *Scmv2* locus in three of the susceptible recombinants, Nos.70, 683 and 1045, was identical to that in the susceptible parent Zheng58, and thus they could not be used to define the *Scmv1* locus. The four resistant RILs, Nos. 413, 966, 246, and 841, together with one susceptible RIL No. 1012 had the resistance allele as Chang7-2 at the *Scmv2* locus and thus could be used to fine-map the *Scmv1* locus. The four resistant RILs had the Chang7-2 donor region upstream of the marker B4, and one susceptible RIL No. 1012 had the Chang7-2 donor region downstream of the marker SNP4. These data support that the *Scmv1* locus is located upstream of the marker B4 (Figure 2A). The mapped *Scmv1* region in the Zheng58×Chang7-2 RIL population spanned the 112.39-kb *Scmv1* region revealed by the near-isogenic F_2_ population and was thus of no value to further narrow down the *Scmv1* locus.

Among the nine recombinants screened from the RIL population X178×HuangC, two displayed the same high resistance to SCMV as seen in the resistant parent X178, whereas another seven were as susceptible to SCMV as the parent HuangC. Three highly susceptible RILs, Nos. 1779, 1553, and 1746, carry the HuangC susceptibility allele at the *Scmv2* locus. They were thus excluded from fine-mapping of the *Scmv1* locus. Four RILs, Nos. 1900, 1876, 2041, and 2062, had the X178 resistance allele at the *Scmv2* locus. However, they were all susceptible to SCMV. This finding indicates the absence of the X178 resistance allele at the *Scmv1* locus for these four RILs. Among them, Nos. 1876 and 1900 RILs had the donor HuangC region downstream of the marker R1-2, and Nos. 2041 and 2062 RILs had the donor HuangC region upstream of the marker SNP3, suggesting the *Scmv1* locus is confined by the markers R1-2 and SNP3. Two highly resistant RILs, Nos. 2009 and 1523, carried the X178 resistance allele at the *Scmv2* locus and their *Scmv1* regions were flanked by the markers 579P4 and B4. Thus, the *Scmv1* locus can be narrowed down into a 59.21-kb region flanked by the markers 579P4 and SNP3 (Figure 2B), located within the 112.39-kb interval defined by the near-isogenic F_2_ population.

### Predicting putative genes within and around the mapped *Scmv1* region

**Figure 3:**
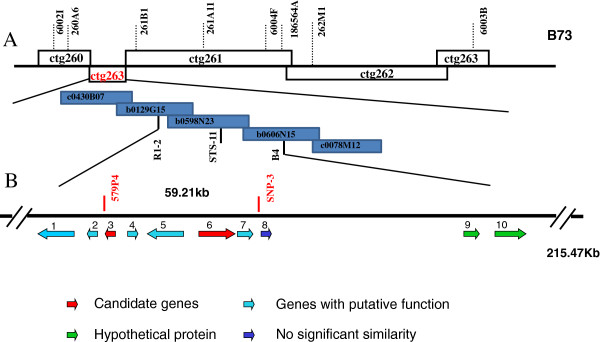
**Integration of the mapping results from three populations. (A)** The combined mapping of *Scmv1*. The revised order of contigs on the *Scmv1* region is #260-#263-#261-#262-#264 (ctg263 in red is where *Scmv1* located ). *Scmv1* was finally assigned to a 59.21-kb region flanked by markers 579P4 and SNP3. **(B)** Gene prediction of a 215.47-kb B73 sequence covering the targeted region: each arrow stands for a predicted gene, the head represents gene orientation and the length of each arrow corresponds to gene length (detailed information listed in Table 3).

A 215.47-kb sequence flanked by the markers B4 and R1-2 was retrieved from http://www.maizesequence.org to identify candidate *Scmv1* genes. There are still seven gaps left in this region, indicating some missing sequences. Transposable elements account for 55.27% of this region, of which 82.28% are retrotransposons, and 99.87% of the retrotransposons are long terminal repeat elements (data not show). We used Fgenesh software (http://linux1.softberry.com/berry.phtml, version 2.6) to predict ten putative genes from the masked sequence, including seven genes with putative functions, two hypothetical genes, and one gene without significant similarity to known genes (Figure 3C). No typical resistance (*R*) gene was found in the *Scmv1* region. Analysis of possible functions for the seven predicted genes with Gene Ontology (GO) and Kyoto Encyclopedia of Genes and Genomes (KEGG) annotation indicated three of the genes were likely to confer resistance to SCMV (Table 3).

**Table 3:** Analysis of genes predicted from the repeat-masked sequence between the 215.47-kb interval of the markers B4/R1-2

**Predicted gene**	**Length of gene**	**First hit**	**E-value**	**Query coverage**	**KEGG entry**
1	403aa	putative cycloartenol synthase	1.00E-129	76%	AT2G07050
2	137aa	60S ribosomal protein L24	7.00E-04	16%	AT3G53020
3*	119aa	thioredoxin H-type	4.00E-64	100%	AT5G39950
4	239aa	putative transposable element	2.00E-29	89%	AT3G22220
5*	360aa	putative cycloartenol synthase	1.00E-74	60%	AT2G07050
6	618aa	sucrose synthase2	4.00E-52	36%	AT4G02280
7	342aa	salT gene product	3.00E-20	100%	AT1G19715
8	93aa	no significant similarity	NA	NA	NA
9	330aa	hypothetical protein cauri_1913	3.30E+00	53%	NA
10	455aa	hypothetical protein	2.00E-107	98%	NA

*stands for candidates. First hit, E-value, and Query coverage were BLASTP results of predicted genes from NCBI. KEGG entry is the search word for pathway information about the predicted genes based on GO blast search results. NA means not available.

Two of the predicted genes are cycloartenol synthase1-like (*CAS1-like*) genes with high sequence similarity to genes from *Arabidopsis thaliana.* The first had the coverage of 76% and E-value of 1.00E-129, whereas the second had the coverage of 60% and E-value of 1.00E-74. These two *CAS1-like* genes have 74.18% similarity (204/275), with an E value of 2.00E-117, although they differ in the lengths of their amino-acid sequences. We designated the larger gene (403 residues) as *CAS1-like-1*, and the smaller one (360 residues) as *CAS1-like-2*. The other candidate gene, *Zmtrx-h*, encodes a putative 119-residue protein that is highly similar to thioredoxin (100% coverage; E = 4.00E-64), an oxidoreductase that acts on sulfur groups of donors, using disulfide as an acceptor.

Integration of the mapping results indicated that *Scmv1* localizes to a 59.21-kb region flanked by 579P4 and SNP3 (Figure 3B). Of the five predicted genes located in this region, *CAS1-like-2* and *Zmtrx-h* are more likely than the other three genes to confer resistance to SCMV.

### Candidate gene–based association mapping of the *Scmv1* locus

**Figure 4:**
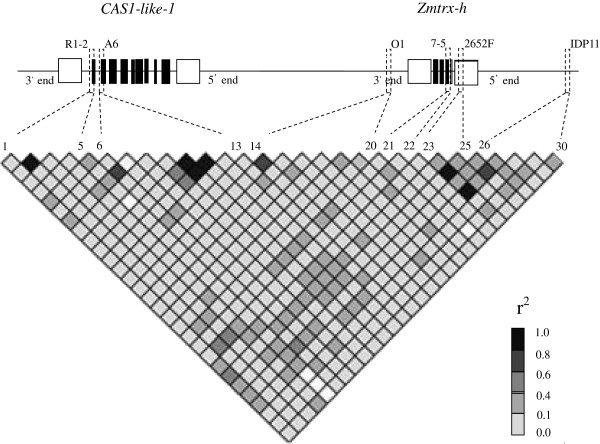
**Gene structure and LD pattern of the *Scmv1 *region across 94 maize inbred lines.** All polymorphic sites with MAF ≥ 0.05 were used. In the gene diagram, filled black boxes represent exons, and open boxes indicate the UTRs. Dash-dot boxes mark the regions sequenced in this study. Polymorphic sties 1 to 5 are from the sequenced PCR product R1-2, 6 to 13 from A6, 14 to 20 from O1, 21 to 22 from 7–5, 23 to 25 from 2652F, and 26 to 30 from IDP11. The polymorphic sites and their locations on the gene diagram are connected by Dash-dot lines.

To verify the candidate region and possibly refine the analysis by identifying quantitative trait nucleotide polymorphisms, we conducted an association mapping study using a panel of 94 inbred lines (Additional file 2), which were divided into six subgroups based on 70 SSR markers (Additional file 3). Multiple sequence alignment analysis identified 30 polymorphisms in six informative amplicons. Linkage disequilibrium (LD) analysis revealed lower r^2^ between these polymorphisms (Figure 4), with 91.03% of the r^2^ < 0.20. Two (single nucleotide polymorphism) SNP sites, S454 and S126, separately located on the 3′-end and intron of *Zmtrx-h* were significantly associated with SCMV resistance (*P <* 0.05; Table 4 and Additional file 4). Nonetheless, a low r^2^ value of 0.01 indicated two independent functional variants.

**Table 4:** **Associations between SCMV resistance and polymorphic sites in the *Scmv1 *** **genome region**

**Sites**	**Location**	**Alleles**^**a**^	**Frequency**	**P-value**		**FAP1360A/F7**^**d**^
				**GLM**^**b**^	**SMA**^**c**^	
S454	3^,^-end of *Zmtrx-h*	T/**G**	15/50	0.0145	0.0039	**G**/T
S126	Intron of *Zmtrx-h*	**T**/C	5/68	0.0371	0.0281	C/C
PAV	Bin 6.01	0/**1**	19/75	9.56E-4	2.07E-5	**1**/**1**

S454 was identified from the marker O1, while S126 was identified from the marker 7–5.

^a^Letters (or number) in bold indicate the favourable alleles.

^b^P-value was calculated using GLM in TASSEL 2.0.

^c^P-value was calculated using single marker analysis.

^d^The alleles of inbred lines FAP1360 and F7.

Given that 19 of the 94 maize inbred lines, including Mo17, could not be amplified by polymerase chain reaction (PCR) using any of the 17 primer pairs scattered throughout the *Scmv1* region, we were able to identify a large PAV covering the *Scmv1* region. This PAV was verified by Springer et al. [23], who reported a 2.6-Mb region on chromosome 6 including the *Scmv1* region present in the B73 genome but not in Mo17. Strikingly, all of the 19 inbred lines were susceptible to SCMV, resulting in a strong association with SCMV resistance (*P* = 9.96E-4).

## Discussion

Precise phenotypic evaluation is crucial for marker-trait association analysis [24-27], especially for those traits with incomplete penetrance and phenotypic plasticity. Apart from being influenced by two major genes, resistance of maize to SCMV is also affected by genetic background and various environmental factors [13,17,18]. To obtain reliable phenotypic data, we developed a F_2_ mapping population from a near-isogenic pair where the resistance allele was fixed at the *Scmv2* locus, and only *Scmv1* was segregating [28]. Furthermore, selection of only susceptible individuals from the F_2_ population for fine-mapping of the *Scmv1* locus reduced the possibility of misclassification, as heterozygotes tend to be more plastic in their response to SCMV inoculation.

Advances in multiple genomic platforms and analytical methods allow fine-mapping and cloning of QTL responsible for important agronomic traits [29]. In the current study, development of polymorphic markers in the 112.39-kb *Scmv1* region was a challenge due to highly repetitive sequences. We thus sequenced the positive BAC clones from the 1145, FAP1360A, and Huangzao4 BAC libraries to mask the repetitive sequences at the *Scmv1* locus, in order to reveal single/low-copy sequences for marker development. Generally, re-sequencing of more maize inbred lines [30] and the availability of additional publicly accessible sequence information (such as for Mo17: http://www.phytozome.net/maize.php; and Palomero: http://www.palomerotoluqueno.org/index.php) will facilitate the development of markers in targeted genome regions. During the process of fine-mapping, we found that the published order of BAC contigs (http://www.maizesequence.org) for the *Scmv1* region is incorrect. The BAC-based B73 whole-genome physical map suggests that the adjacent BAC contigs #260 and #261 should be physically close to one another. However, the genetic distance is so large that none of the recombinants within the umc1018/umc1753 interval could be further resolved by the newly developed markers based on the contig #261. Only the markers developed from the contig #263 could resolve the recombinants. This indicates that contig #263 is located between contigs #260 and #261, and that the correct contig order must be #260-#263-#261-#262-#264 (Figure 3A). This correct contig order has been released (http://www.maizesequence.org).

Searching for recombinants with an unambiguous phenotype is crucial for fine-mapping. Thus, we used only highly susceptible individuals in the F_2_ mapping population for fine-mapping, which allowed us to fine-map the *Scmv1* locus into a 112.39-kb region flanked by R1-2 and STS-11. From the fact that the *Scmv1* locus has been repeatedly detected [8,12,14,21], we inferred that *Scmv1* is likely present in various resistant inbred lines. Based on this hypothesis, two RIL populations were used to fine-map the *Scmv1* locus into a 59.21-kb region flanked by the markers 579P4 and SNP3.

The public B73 sequence suggested that two predicted genes, the *CAS1*-like homolog and *Zmtrx-h* gene, are the most likely candidates for *Scmv1* owing to their potential roles in disease defense response [31,32], although the potential influence of hypothetical proteins in the *Scmv1* region on SCMV resistance cannot be excluded. Comparison with the complete *CAS1* gene from *Arabidopsis thaliana*, which encodes 759 residues, suggests that the putative *CAS1-like* gene may be partial gene containing the TERPENE_SYNTHASES motif. CAS1 is involved in biosynthesis of steroids and non-steroidal secondary metabolites, such as campesterol and stigmasterol [31]. Campesterol is the precursor of brassinosteroids, an important class of plant hormones that functions in cellular signal transduction, whereas the accumulation of stigmasterol stimulates an important plant metabolic process that occurs following bacterial infection of leaves [32]. Given that phenotypic variance associated with changes in the *Scmv1* region are influenced by the stage of plant development [19], it remains to be established whether the situation with *Scmv1* closely resembles that of *Hm2*. a truncated duplicate of *Hm1*. Compared to *Hm1*, *Hm2* preferentially functions in adult plants, conferring resistance against the leaf spot and ear mold disease caused by *Cochliobolus carbonumrace 1*[33,34].

Thioredoxin is a master regulator of cellular redox status [35], with h-type thioredoxin reported to function in defense responses to viruses [36] and fungi [37]. Given that the *Zmtrx-h* protein in the *Scmv1* region lacks the conserved WC(G/P)PC motif, which is essential for redox protein activities, it seems unlikely that *Zmtrx-h* affects cellular redox status. Hence, additional investigation of the roles of the three candidate genes is required to uncover the mechanism of SCMV resistance.

Linkage and association analysis are two prevalent approaches to map genes or QTL. Both can be used in a complementary manner to dissect the genetic basis of traits of interest [38,39], as well as to fine-map causative variants of targeted QTL [40]. Six pairs of primers were used to test the association of specific stretches of sequence in the *Scmv1* region with SCMV resistance. General linear model (GLM) analysis highlighted three polymorphisms associated with SCMV resistance. The significant association between the PAV and SCMV resistance validates the role of the *Scmv1* region with disease resistance, whereas the association between two other SNPs and SCMV resistance provides statistical support for *Zmtrx-h* as the primary candidate for *Scmv1*. Moreover, these two SNP located in the intron and 3′-end of *Zmtrx-h*, respectively, seemingly having an influence on the gene’s expression. The *CAS1-like-2* gene cannot yet be excluded, as there was no primer pair available in the vicinity of it. Therefore, a complementary functional test via transformation is still needed to identify which candidate gene, if any, defines *Scmv1*. These transgenic experiments are underway in our lab.

Broad-spectrum resistance (BSR), resistance against two or more types of pathogen species or the majority of races of the same pathogen species [41], is essential to improve the resistance of crops to various diseases. The quantitative resistance gene *Lr34* cloned recently confers resistance to leaf rust, stripe rust, powdery mildew and various other diseases of wheat [42]. The evidence for the presence of multiple disease resistance genes in a maize association population [43] revealed that quantitative BSR can be conferred by a single gene. The *Scmv1* region has been reported to associate with resistance to other members of the *Potyviridae* family, including WSMV and MDMV [44,45]. The near isogenic line F7^RR/RR^, which was used as a resistant parental line in this study, was also found to be resistant to MDMV and ZeMV [28]. According to a model proposed by Kou [46], a single gene conferring BSR may function in either basal-resistance pathways, in overlapping pathways that confer race-specific resistances, or at sites of cross-talk between different defense pathways. Given that both candidate genes have putative roles in basal resistance, *Scmv1* likely confers BSR, which would be advantageous in environments where plants are threatened by multiple pathogens. However, this speculation remains to be verified by artificial inoculation of transgenic plants and recombinants with the appropriate range of pathogens.

## Conclusion

We have fine-mapped *Scmv1* to a 59.21-kb region through integrating mapping data from different populations. This provides indispensable data for initiating molecular breeding to improve SCMV resistance in maize, a crop that is critical to world food security. We identified two candidates, of which the *Zmtrx-h* gene seems the most promising. Map-based cloning of *Scmv1* and transgenic analyses will shed light on the molecular mechanism of this important plant-virus interaction.

## Methods

### Populations used for fine-mapping

The F7 near-isogenic lines, F7^RR/RR^, F7^RR/SS^, and F7^SS/RR^, derived from two early-maturing European maize inbred lines, FAP1360A (completely resistant to SCMV) and F7 (highly susceptible to SCMV), have been developed as described by Ingvardsen [17] (Additional file 5). A total of 2,200 F_2_ plants derived from the cross of F7^RR/RR^×F7^RR/SS^ were collected as a mapping population for fine-mapping of *Scmv1*. Initially, a panel of 177 plants, randomly selected from the F_2_ mapping population, was used as an unselected mapping population to delimit the *Scmv1* region. Each plant was mechanically inoculated with SCMV twice and scored at a weekly interval to evaluate resistance to SCMV. Additional markers in the *Scmv1* region were developed to genotype the unselected F_2_ population. In parallel, the remaining 2,023 F_2_ plants were investigated for their resistance to SCMV, resulting in the identification of 510 plants with typical SCMV symptoms indistinguishable from their susceptible parent F7. These 510 susceptible F_2_ plants, together with 28 recombinants from the unselected F_2_ mapping population, formed the F_2_ sub-population for fine-mapping of *Scmv1*.

### Validation of the *Scmv1* locus in two RIL populations

Two single-seed descent F_6_ RIL populations were employed to validate and further fine map the *Scmv1* locus. One was developed from the cross between Zheng58 (susceptible to SCMV) and Chang7-2 (resistant to SCMV), a commercial hybrid (ZD958) that is currently widely cultivated in China. The other RIL population was derived from the cross between X178 (completely resistant to SCMV) and HuangC (highly susceptible to SCMV), another commercial hybrid (ND108) widely grown in China. A total of 2,206 F_6_ RILs (1,285 from Zheng58×Chang7-2 and 921 from X178×HangC) were planted in the experimental station at Shangzhuang, Beijing, during the summer of 2010. All RILs were genotyped using the markers R1-2 and B4 flanking the *Scmv1* locus, as well as a co-segregating marker DJF004 at the *Scmv2* locus [17]. For each RIL population, four sets of 30 RILs, which differed at the *Scmv1* or *Scmv2* locus or both loci, were separately selected for phenotypic evaluation, whereas all recombinants between R1-2 and B4 were investigated for their SCMV resistance.

### Artificial inoculation

Seeds were sown in a soil/vermiculite mixture in 0.80-L pots maintained under greenhouse conditions. Virus inocula (Chinese isolate) were prepared from the SCMV-infected susceptible F7 plants. Young leaf tissue with typical mosaic symptoms was homogenized in five volumes of 0.01 M sodium phosphate buffer, pH 7.0. Carborundum was added to the sap to facilitate rub-inoculation. All leaves of each plant were rub-inoculated at the three- to four-leaf stage. One week after inoculation, all the symptomless plants were re-inoculated to avoid any escape. During sap preparation and mechanical inoculation, the inocula were put on ice to maintain low-temperature conditions.

For phenotypic evaluation of inbred lines and selected RILs, 12 kernels of each line were planted in four 0.80-L seeding pots. Whereas 10 plants were rub-inoculated with virus inoculum, two plants were not inoculated. These RILs were evaluated along with their parent lines as positive and negative controls for all tests. RILs with the same symptoms (*P* > 0.05, t-test) as the susceptible parent or the resistant parent were regarded as susceptible (S) or resistant (R). Three independent experiments were performed during September 2010 to July 2011 to ensure accurate phenotyping.

### DNA extraction and molecular analyses

Immature leaf samples were harvested from each individual and immediately frozen in liquid nitrogen. DNA was extracted using the SDS method described by Murray and Thompson [47].

Apart from new markers developed from public BAC sequences, all SSR markers were retrieved from the Maize Genetics and Genomics Database (http://www.maizegdb.org) (Additional file 1) and synthesized by Invitrogen (Beijing, China). To design specific markers for fine-mapping, we first anchored the QTL in bin 6.01 to the B73 BAC-based whole-genome physical map (http://www.maizesequence.org/) using flanking SSR markers. We next extracted BACs, expressed sequence tags, and Indel polymorphic sequences (http://www.ncbi.nlm.nih.gov/; http://magi.plantgenomics.iastate.edu/browseMarkers.do) available in the targeted region to develop new polymorphic markers (SSR, STS, SNP, and CAPS). The retrieved sequences were analyzed with RepeatMasker (http://www.repeatmasker.org/cgi-bin/WEBRepeatMasker) followed by BLAST analysis against maize high-throughput genome sequence and genome sequence survey to remove redundant sequences [48]. All primers for the single/low-copy sequences were designed using the software PRIMER 5.0 with the criteria that the primer length should be 20 nucleotides, with a 40–60% GC content, no secondary structure, and no consecutive tracts of the same nucleotide. The microsatellite search tool SSRHunter1.3 [49] was used to mine SSR sequences of 4–6 tandem copies of >2 base pairs.

For PCR-mediated detection of SSR and STS markers, reactions were prepared in a 10-μL reaction volume containing 100 ng of genomic DNA, 1 μL of 10× PCR buffer, 0.2 mM of each dNTP, 0.2 μM of each forward and reverse primer, and 1 U of Taq polymerase. To detect SNP and CAPS markers, 50 μL reactions contained 300 ng of template DNA, 5 μL of 10× PCR buffer, 1 mM of each dNTP, 1 μM of each forward and reverse primer, and 5 U Taq polymerase (Beijing Transgen Biotech Co.LTD). All PCR reactions were performed using a PTC-200 Peltier Thermal Cycler (MJ Research Inc., Canada) with particular annealing temperature and elongation duration adapted to each reaction. The PCR products were subjected to electrophoresis using either 1% agarose gels (visualized using a gel imaging system from Bio-Rad Laboratories Inc., USA) or 6% polyacrylamide gels (amplification products were visualized by silver staining). SNP markers were sequenced at the Beijing Genomic Institute.

### Analysis of candidate genes

To predict candidate genes, we retrieved the DNA sequence of the *Scmv1* region from the B73 whole-genome sequence (http://www.maizesequence.org) for candidate gene prediction. The repetitive sequence in the *Scmv1* region was analyzed by RepeatMasker version 3.3.0 (http://www.repeatmasker.org). We used the Fgenesh program (http://linux1.softberry.com/berry.phtml, version 2.6) to predict genes present in the single/low-copy *Scmv1* region. For functional analysis, the deduced amino-acid sequence of each putative gene was compared against the “nr” database via BLASTP on National Center of Biotechnology Information website (http://blast.ncbi.nlm.nih.gov/). Detailed GO and KEGG annotation was applied to the gene sequences predicted by BLAST searches (http://www.geneontology.org/ and http://www.genome.jp/kegg/).

### Association mapping of the *Scmv1* region

A total of 94 maize inbred lines were phenotyped in two distinct experiments (October 2007, May 2010) under greenhouse conditions using the same artificial inoculation procedure as RIL populations. The same 94 inbred lines were genotyped using a set of 70 SSR markers that were evenly distributed throughout the maize genome (Additional file 6). Based on the B73 sequence and using Primer 3 (http://frodo.wi.mit.edu/) or Premier 5.00 (PREMIER Biosoft International, USA), we designed 17 pairs of PCR primers (Additional file 7) flanking the three candidate genes. We used these primers to first screen for polymorphisms between F7, FAP1360A, Zheng58, Chang7-2, HuangC, and X178. Most primer pairs were discarded because of unreliable amplification. Six primer pairs with polymorphic products were used to amplify these 94 inbred lines to identify potential polymorphic sites. Of these six polymorphic amplicons, two and three are located in the vicinity of *CAS1-like-1* and *Zmtrx-h*, respectively, whereas no primer pair around *CAS1-like-2* was available. Nucleotide sequences alignments were done using the multiple sequence alignment software MUSCLE [50] and subsequently refined manually. Q was inferred using STRUCTURE 2.2 [51,52] with 70 SSR markers. Five independent runs were performed setting the number of populations (k) from 1 to 10, burn in time and MCMC (Markov Chain Monte Carlo) replication number both to 500,000, and a model for admixture and correlated allele frequencies. The k value was determined by LnP(D) in STRUCTURE output and an ad hoc statistic Δk based on the rate of change in LnP(D) between successive k. Lines with membership probabilities ≥ 0.75 were assigned to corresponding clusters; lines with membership probabilities < 0.75 were assigned to a mixed group. Groups were further subdivided into subgroups using a similar methodology. The runs most consistent with breeder’s knowledge about pedigree were used to assign lines into clusters. The levels of LD between two sites were calculated using TASSEL2.0 [53]. The associations between the extracted polymorphic sites with Minor Allele Frequency (MAF) ≥ 0.05 and SCMV resistance were carried out using GLM in TASSEL2.0.

## Abbreviations

BAC: Bacterial artificial chromosome; BSR: Broad-spectrum resistance; CAS: Cycloartenol synthase1-like; GO: Gene ontology; K: Kinship; KEGG: Kyoto encyclopedia of genes and genomes; LD: Linkage disequilibrium; MAF: Minor Allele Frequency; JGMV: Johnson grass mosaic virus; GLM: General linear model; PCR: Polymerase chain reaction; Q: Population structure; PAV: Presence/absence variations; QTL: Quantitative trait loci; RIL: Recombinant inbred line; SCMV: Sugarcane mosaic virus; SNP: Single nucleotide polymorphism; SrMV: Sorghum mosaic virus; SSR: Simple sequence repeat; WSMV: Wheat streak mosaic virus; ZeMV: Zea mosaic virus.

## Competing interests

The authors declare that they have no competing interests.

## Authors’ contributions

LJ, QL, CRI and UKF performed mapping experiment in segregating population. BW and JL provided two RIL populations. YT, QL, carried out fine-mapping in these two RIL populations. YZ, RZ and YT completed the association mapping work. MX and TL supervised the research, designed the experiments and were involved in data analysis. YT wrote the manuscript draft, MX and TL edited and revised the manuscript. All authors read and approved the final manuscript.

## Supplementary Material

Additional files 1 **The list of public SSR markers for mapping of *Scmv1.***
Click here for file

Additional files 2 The 94 maize inbred lines and their resistance performance to SCMVR means resistance to SCMV, while S means susceptible to SCMV. Click here for file

Additional files 3 **Population structure of 94 maize inbred lines estimated using 70 SSRs.** Population structure was assessed by STRUCTURE. Each individual is represented by a vertical bar, partitioned into colored segments with the length of each segment representing the proportion of the individual’s genome from k = 6 groups. For all classes, a given group is represented: red, REID; Blue, P; green, LAN; Yellow, TSPT; pink, Z330; turquoise, OTHER. Click here for file

Additional files 4 **Associations between SCMV resistance and polymorphic sites in the *Scmv1 *genome region.** a: P-value calculated using GLM. #: polymorphic sites associated with SCMV resistance. Click here for file

Additional files 5 **The procedure of developing the *Scmv1 *** **mapping population.** Click here for file

Additional files 6 List of SSR markers for structure and kinship analysis. Click here for file

Additional files 7 **Primers designed for association mapping.** *: The PCR products amplified by these primers contained polymorphic sites that could be used for association mapping. Click here for file
