# Supplementary material for: Combined linkage and association mapping reveals candidates for Scmv1, a major locus involved in resistance to sugarcane mosaic virus (SCMV) in maize
Source: BMC Plant Biol. 2013 Oct 18;13:162. doi: 10.1186/1471-2229-13-162 (PMC4016037; doi:10.1186/1471-2229-13-162)
Supplement: Additional files 1 — The list of public SSR markers for mapping of Scmv1. [file 1471-2229-13-162-S1.docx]

**Table S1**.Public markers for mapping of *Scmv1*

| Name | Forward Primer | Reverse Primer | Annealing temperature(℃) | Marker information |
| --- | --- | --- | --- | --- |
| bnlg1043 | TTTGCTCTAAGGTCCCCATG | CATACCCACATCCCGGATAA | 60 | SSR |
| umc2309 | GTCGAAATTCCTGACACAAAAAGG | CTGTGTTTTGTGTATTAGCGCCAG | 60 | SSR |
| umc2208 | CTTGATCGACATTTCGTTATGCTG | ACCAAATCAAACGAAGAAAAGTGG | 60 | SSR |
| phi126 | TCCTGCTTATTGCTTTCGTCAT | GAGCTTGCATATTTCTTGTGGACA | 60 | SSR |
| bnlg161 | GCTTTCGTCATACACACACATTCA | ATGGAGCATGAGCTTGCATATTT | 60 | SSR |
| umc2068 | CCGCTCCTTCTCCTCCTCATC | GGAACTCCTCGAGCGTGAGC | 60 | SSR |
| umc1018 | GAACGGATATTGGAACCTGTGC | GTGCACGGTGTCGTACTTGAAC | 60 | SSR |
| umc1196 | CGTGCTACTACTGCTACAAAGCGA | AGTCGTTCGTGTCTTCCGAAACT | 60 | SSR |
| umc1825 | GCGCTGCCAACTGTATCTTTATCT | AAGTTACTCACGGTGCAGAGTTCC | 60 | SSR |
| umc1753 | AAGATCTTGCTCCGTTTCCTCTCT | TTCAGATGCAAATCTCTTTTCGCT | 60 | SSR |
| umc1600 | CGATCAGTGCGTGGAGAGTA | TAGGCATGCATTGTCCATTG | 60 | SSR |
| bnlg1432 | AAAGCAAACAAACAATGGGC | TGCGTGCAGTGACATATTCA | 60 | SSR |
| bnlg1165 | CGCTTGCATCATCTCAAGAA | TTCAAGTTTAGCCACCCACC | 58 | SSR |
| umc2312 | GCTGATGAGCTCGCCATTCT | ACCCCATCTCAGTCTCACTCACTT | 58 | SSR |
| bnlg1867 | CCACCACCATCGTAGGAGTT | CAGTACACAGCAGGCAGCTC | 58 | SSR |
| umc1229 | AAACTTCTCCCCCGCAGTTC | CACCAACTCCACCACGTTCC | 62 | SSR |
| phi077 | GAGAAGAGGATCAGGTTCGTTCCA | CGCGTTGTACATCTTGCCTGCTT | 62 | SSR |
