# Supplementary material for: Combined linkage and association mapping reveals candidates for Scmv1, a major locus involved in resistance to sugarcane mosaic virus (SCMV) in maize
Source: BMC Plant Biol. 2013 Oct 18;13:162. doi: 10.1186/1471-2229-13-162 (PMC4016037; doi:10.1186/1471-2229-13-162)
Supplement: Additional files 2 — The 94 maize inbred lines and their resistance performance to SCMVR means resistance to SCMV, while S means susceptible to SCMV. [file 1471-2229-13-162-S2.docx]

**Table S2**. 94 maize inbred lines and corresponding phenotype

| No. | Name | Phenotype | No. | Name | Phenotype | No. | Name | Phenotype |
| --- | --- | --- | --- | --- | --- | --- | --- | --- |
| 1 | 698-3 | R | 32 | Ji853 | R | 63 | 1145 | R |
| 2 | Chang7-2 | R | 33 | B73 | S | 64 | Huangzao4 | R |
| 3 | Zheng58 | S | 34 | Tian77 | S | 65 | Lian87 | S |
| 4 | Yu374 | S | 35 | FEB-48 | S | 66 | Hai9-21 | R |
| 5 | 4F1 | S | 36 | P178 | R | 67 | Jiao3 | R |
| 6 | Dan598 | R | 37 | HuangC | S | 68 | Ay420 | S |
| 7 | LX9801 | R | 38 | Ji53 | R | 69 | A50 | S |
| 8 | Jiao51 | S | 39 | K10 | S | 70 | Zi341 | S |
| 9 | S37 | R | 40 | Qi846 | S | 71 | Jun971 | R |
| 10 | Ji69 | S | 41 | Zong31 | S | 72 | Ay3566 | R |
| 11 | Dan340 | S | 42 | Qi319 | R | 73 | 6256 | S |
| 12 | 5237 | R | 43 | Zheng32 | S | 74 | 6407 | S |
| 13 | Xi502 | R | 44 | Huotanghuang | S | 75 | 7416 | S |
| 14 | K22 | R | 45 | Huangyesi | R | 76 | 7397 | R |
| 15 | Dan599 | R | 46 | U8112 | S | 77 | 7368 | S |
| 16 | Sheng137 | R | 47 | 5003 | R | 78 | 7364 | S |
| 17 | Q1537 | S | 48 | Zheng22 | S | 79 | 7296 | R |
| 18 | Qi205 | S | 49 | 7884 | R | 80 | 7286 | R |
| 19 | K12 | R | 50 | 515 | S | 81 | 7314 | S |
| 20 | Nan213 | R | 51 | 52106 | S | 82 | 9508 | R |
| 21 | P138 | R | 52 | 444 | S | 83 | Siyi | R |
| 22 | Ji4112 | R | 53 | 122 | S | 84 | Va35 | S |
| 23 | 5311 | S | 54 | Ye107 | R | 85 | Dong46 | S |
| 24 | 8701 | R | 55 | Zi330 | S | 86 | Longkang11 | S |
| 25 | Lv28 | S | 56 | Mo17 | S | 87 | Chang3 | S |
| 26 | Wu109 | S | 57 | 478 | S | 88 | 8001 | S |
| 27 | Dan9046 | R | 58 | F7 | S | 89 | Danhuang02 | S |
| 28 | BT1 | R | 59 | H99 | R | 90 | Chang72 | S |
| 29 | Ye832 | S | 60 | 10940 | S | 91 | Yan414 | S |
| 30 | 1121 | R | 61 | A188 | S | 92 | Q1261 | S |
| 31 | Tie7922 | S | 62 | FAP1360A | R | 93 | Ji63 | S |
|  |  |  |  |  |  | 94 | Ji842 | S |

R means incompatible with SCMV, while S means compatible with SCMV.
