# Supplementary figures and images for: Combined linkage and association mapping reveals candidates for Scmv1, a major locus involved in resistance to sugarcane mosaic virus (SCMV) in maize
Source: BMC Plant Biol. 2013 Oct 18;13:162. doi: 10.1186/1471-2229-13-162 (PMC4016037; doi:10.1186/1471-2229-13-162)

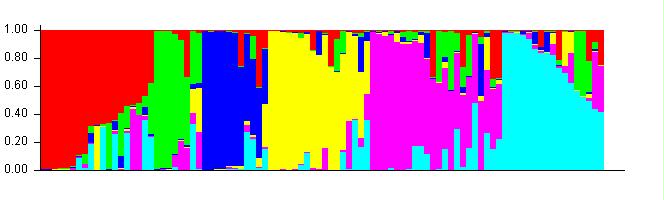


REID LAN P TSPT Z330 OTHER

Figure S1

Supplement: Additional files 3 — Population structure of 94 maize inbred lines estimated using 70 SSRs. Population structure was assessed by STRUCTURE. Each individual is represented by a vertical bar, partitioned into colored segments with the length of each segment representing the proportion of the individual’s genome from k = 6 groups. For all classes, a given group is represented: red, REID; Blue, P; green, LAN; Yellow, TSPT; pink, Z330; turquoise, OTHER. [file 1471-2229-13-162-S3.docx]
