# Supplementary material for: Combined linkage and association mapping reveals candidates for Scmv1, a major locus involved in resistance to sugarcane mosaic virus (SCMV) in maize
Source: BMC Plant Biol. 2013 Oct 18;13:162. doi: 10.1186/1471-2229-13-162 (PMC4016037; doi:10.1186/1471-2229-13-162)
Supplement: Additional files 4 — Associations between SCMV resistance and polymorphic sites in the Scmv1 genome region. a: P-value calculated using GLM. #: polymorphic sites associated with SCMV resistance. [file 1471-2229-13-162-S4.docx]

**Table S5**, Associations between SCMV resistance and polymorphisms in the *Scmv1* genome region

| Sites | Location | Alleles | Frequency | P-value^a^ | Primer |
| --- | --- | --- | --- | --- | --- |
| #S126 | Intron of *Zmtrx-h* | T/C | 5/68 | 0.0371 | 7-5 |
| S261 | Intron of *Zmtrx-h* | G/C | 8/65 | 0.9059 | 7-5 |
| S153 | Exon of *CAS1-like-1* | T/C | 5/56 | 0.4877 | A6 |
| S155 | Exon of *CAS1-like-1* | C/T | 7/61 | 0.1216 | A6 |
| S374 | Exon of *CAS1-like-1* | C/A | 11/57 | 0.3075 | A6 |
| S375 | Exon of *CAS1-like-1* | G/C | 11/58 | 0.3564 | A6 |
| S376 | Exon of *CAS1-like-1* | A/G | 11/58 | 0.3564 | A6 |
| I151 | Exon of *CAS1-like-1* | 0/4/2 | 6/8/55 | 0.2337 | A6 |
| I373 | Exon of *CAS1-like-1* | 0/1 | 8/60 | 0.3935 | A6 |
| S266 | Bin 6.01 | G/T | 5/53 | 0.4305 | IDP11 |
| I81 | Bin 6.01 | 0/8 | 24/42 | 0.1633 | IDP11 |
| I181 | Bin 6.01 | 0/2 | 16/50 | 0.6002 | IDP11 |
| I251 | Bin 6.01 | 0/4 | 24/31 | 0.7922 | IDP11 |
| I255 | Bin 6.01 | 5/2/3/4 | 7/9/12/31 | 0.5235 | IDP11 |
| S97 | 3’ end of *Zmtrx-h* | T/C | 17/56 | 0.7891 | O1 |
| S152 | 3’ end of *Zmtrx-h* | A/G | 17/57 | 0.8047 | O1 |
| S231 | 3’ end of *Zmtrx-h* | A/G | 20/54 | 0.4055 | O1 |
| S396 | 3’ end of *Zmtrx-h* | A/G | 25/49 | 0.4091 | O1 |
| S397 | 3’ end of *Zmtrx-h* | T/C | 9/65 | 0.1553 | O1 |
| #S454 | 3’ end of *Zmtrx-h* | G/T | 15/50 | 0.0145 | O1 |
| S456 | 3’ end of *Zmtrx-h* | C/G | 5/56 | 0.397 | O1 |
| S121 | Exon of *CAS1-like-1* | A/C | 6/66 | 0.0878 | R1-2 |
| I15 | Exon of *CAS1-like-1* | 4/0 | 34/37 | 0.512 | R1-2 |
| I123 | Exon of *CAS1-like-1* | 0/3 | 5/67 | 0.1747 | R1-2 |
| I219 | Exon of *CAS1-like-1* | 0/5 | 31/42 | 0.7261 | R1-2 |
| I234 | Exon of *CAS1-like-1* | 8/2/4/6 | 5/8/21/35 | 0.42 | R1-2 |
| S47 | 5’UTR of *Zmtrx-h* | T/C | 21/53 | 0.2944 | 2562F |
| S471 | 5’UTR of *Zmtrx-h* | T/C | 7/64 | 0.3952 | 2562F |
| S472 | 5’UTR of *Zmtrx-h* | A/G | 14/59 | 0.7799 | 2562F |
| #PAV | Bin 6.01 | 0/1 | 19/75 | 9.56E-4 | All |

a, P-value was calculated using GLM.

# indicates polymorphism associated with SCMV resistance
