# Supplementary material for: Combined linkage and association mapping reveals candidates for Scmv1, a major locus involved in resistance to sugarcane mosaic virus (SCMV) in maize
Source: BMC Plant Biol. 2013 Oct 18;13:162. doi: 10.1186/1471-2229-13-162 (PMC4016037; doi:10.1186/1471-2229-13-162)
Supplement: Additional files 6 — List of SSR markers for structure and kinship analysis. [file 1471-2229-13-162-S6.docx]

**Table S3．**List of SSR markers for structure and kinship analysis

| **No**. | Locus name | Chr Bin | Repeat Motif | No. | Locus name | Chr Bin | Repeat Motif |
| --- | --- | --- | --- | --- | --- | --- | --- |
| 1 | bnlg1007 | 1.02 | (GA)_15_ | 36 | umc1859 | 6.06 | GA |
| 2 | bnlg439 | 1.03 | TC | 37 | phi299852 | 6.07 | CTG |
| 3 | umc1335 | 1.06 | AG | 38 | umc1127 | 6.08 | GA |
| 4 | umc1147 | 1.07 | (CA)_7_ | 39 | umc1545 | 7 | AAGA |
| 5 | bnlg2331 | 1.11 | ATCC | 40 | umc1066 | 7.01 | / |
| 6 | umc1185 | 2.03 | GC | 41 | umc2160 | 7.01 | AG |
| 7 | umc2129 | 2.07 | GGC | 42 | bnlg1792 | 7.02 | CT |
| 8 | bnlg1940 | 2.08 | (AG)_18_ | 43 | bnlg1380 | 7.02 | GA |
| 9 | phi101049 | 2.1 | GATA | 44 | umc2098 | 7.02 | GCA |
| 10 | bnlg1523 | 3.02 | GA/CT | 45 | umc2057 | 7.02 | GCT |
| 11 | bnlg1452 | 3.04 | TC | 46 | umc2098 | 7.02 | GCA |
| 12 | phi053 | 3.05 | GTAT | 47 | umc1936 | 7.03 | TG |
| 13 | umc1489 | 3.07 | (GCG)_5_ | 48 | phi328175 | 7.04 | GAG |
| 14 | bnlg1754 | 3.09 | CT | 49 | umc1125 | 7.04 | CTCG |
| 15 | umc1136 | 3.1 | GCA | 50 | phi328175 | 7.04 | GAG |
| 16 | phi072 | 4.01 | GTTT | 51 | umc2004 | 8.02 | CT/GT |
| 17 | phi021 | 4.03 | AG | 52 | bnlg2235 | 8.02 | TGG |
| 18 | bnlg490 | 4.04 | TA | 53 | umc1741 | 8.03 | / |
| 19 | bnlg2291 | 4.06 | AGC | 54 | bnlg2082 | 8.03 | CT |
| 20 | umc1051 | 4.08 | CT | 55 | umc1778 | 8.03 | GTC |
| 21 | umc1940 | 4.09 | GCA | 56 | umc1741 | 8.03 | AG |
| 22 | umc1999 | 4.09 | TGC | 57 | umc1309 | 8.05 | / |
| 23 | bnlg589 | 4.1 | GA | 58 | phi080 | 8.08 | GAGAG |
| 24 | umc1496 | 5 | GCA | 59 | umc1933 | 8.08 | CCA |
| 25 | umc2115 | 5.02 | GCCAT | 60 | phi233376 | 8.09 | CGG |
| 26 | umc1705 | 5.03 | TC | 61 | umc2084 | 9.01 | (CTAG)_4_ |
| 27 | umc1429 | 5.03 | AGC | 62 | phi065 | 9.03 | GTGAA |
| 28 | mmc0081 | 5.05 | CT | 63 | umc1492 | 9.04 | GCA |
| 29 | umc1019 | 5.06 | CT | 64 | umc1231 | 9.05 | GA |
| 30 | umc1524 | 5.06 | / | 65 | bnlg1191K7 | 9.06 | TC/GCTA |
| 31 | bnlg2305 | 5.07 | (AG)_21_ | 66 | phi041 | 10 | CAGC |
| 32 | bnlg161 | 6 | AG | 67 | umc1432 | 10.02 | TC |
| 33 | bnlg249 | 6.01 | AG | 68 | bnlg1712 | 10.03 | GA） |
| 34 | bnlg1702 | 6.05 | (CAGCCT)4 | 69 | umc2163 | 10.04 | （AG）28 |
| 35 | bnlg1154 | 6.05 | CT | 70 | bnlg1450 | 10.07 | TC |
