# Supplementary material for: Combined linkage and association mapping reveals candidates for Scmv1, a major locus involved in resistance to sugarcane mosaic virus (SCMV) in maize
Source: BMC Plant Biol. 2013 Oct 18;13:162. doi: 10.1186/1471-2229-13-162 (PMC4016037; doi:10.1186/1471-2229-13-162)
Supplement: Additional files 7 — Primers designed for association mapping. *: The PCR products amplified by these primers contained polymorphic sites that could be used for association mapping. [file 1471-2229-13-162-S7.docx]

**Table S4**, Primers designed for association mapping

| No | Name | Forward primer | Reverse primer |
| --- | --- | --- | --- |
| 1 | *R1-2 | GCGTAGCAATATCCGTGTCA | TTCTGGTGTGAGCTTGAGCC |
| 2 | *A6 | ACCACAAGTCTATGACGCAG | CAGGTACTCGTGCAATATGG |
| 3 | g6full-6 | ATGAAGACGAGAGCACTCAC | TGCGTCATAGACTTGTGGTC |
| 4 | g6full-6-2 | AACCACGATGGCACAGTTAC | TGCGTCATAGACTTGTGGTC |
| 5 | STS-8 | ACCGATTATGTTCCATTGGC | GGTCCGACGCTCACTTCC |
| 6 | Fs-9 | CAGACCTGGTTGCTTAGTCC | AGTGGCAGTGTGGAGTTCAG |
| 7 | g6full-8 | TGTGTCACGTCAGATGTTCC | TCTCATCATAGGCGTCTGTG |
| 8 | *O1 | CGCTCAACATAACCGAGACC | TCCAGAGCCACCATATCACC |
| 9 | D1 | TGTCGTTGTTGATGTGCGTG | TGAAGCGTTCGGTCTCACTG |
| 10 | D2 | CTTGCTATTGCCGAATGGTG | GCCTGTGCCATAGTTGTTGC |
| 11 | *7-5 | ATGTCGTCGTTGACCACAA | GGTCAGAGCGGTTACTAACTC |
| 12 | g6full-14 | TTAGCTGGCACCTAGTCTCC | TTCTGCAAGGTCAAGGTACG |
| 13 | g6full-16 | GGTTGACCTGCCACATTCAG | ACGGAATCACGGAGAACATC |
| 14 | R18-2 | GATCTCGCAATCGTTGTAGC | CTCTCAATGCGGTAACCATC |
| 15 | *IDP-11 | GCAAGGACTGAAGATCGAC | ATGACATGCAACTACAGGCG |
| 16 | STS-12 | CTAACAAGCATGACGATC | AATTGCATCGATAAGCCACC |
| 17 | *2562F | GCCGTATACAGGTCGAGCTT | GGAAGGAAGCCTAACTCGGA |

*indicates primers used for association mapping
